# Establishment of a Sensitive and Visual Detection Platform for Viable *Salmonella* in Wastewater That Combines Propidium Monoazide with Recombinase Polymerase Amplification—CRISPR/Cas12a System

**DOI:** 10.3390/microorganisms13051166

**Published:** 2025-05-21

**Authors:** Jiayin Liang, Xintian Sui, Yan Xu, Xiangqun Zheng, Lu Tan

**Affiliations:** 1 Agro-Environmental Protection Institute, Ministry of Agriculture and Rural Affairs, No. 31 Fukang Road, Nankai District, Tianjin 300191, China; ljyliangjiayin@163.com (J.L.); xuyan@caas.cn (Y.X.); 2 Key Laboratory of Rural Toilet and Sewage Treatment Technology, Ministry of Agriculture and Rural Affairs, No. 31 Fukang Road, Nankai District, Tianjin 300191, China; 3 Guangzhou Urban Planning&Design Survey Research Institute, No. 10 Construction Avenue, Yuexiu District, Guangzhou 510060, China; suixint@foxmail.com; 4 Institute of Environment and Sustainable Development in Agriculture, No. 12 Zhongguancun South Street, Haidian District, Beijing 100081, China

**Keywords:** propidium monoazide, naked-eye detection, recombinase polymerase amplification

## Abstract

Urban sewage, aquaculture wastewater, and medical wastewater are significant reservoirs and transmission sources of *Salmonella*. Rapid detection of *Salmonella* is crucial for effectively reducing the risk of disease transmission and safeguarding public health. Differentiating viable *Salmonella* from inactivated cells presents significant challenges, affecting the accurate assessment of pathogen risks. Moreover, current detection methods face several limitations, including lengthy detection periods, high costs, and limited applicability, underscoring the need for rapid, sensitive, and visual detection diagnostic approaches. In this study, we combined propidium monoazide (PMA) with recombinase polymerase amplification (RPA) and clustered regularly spaced short palindromic repeats (CRISPR)/Cas12a systems to develop a rapid detection system for viable *Salmonella* targeting the *fim*Y gene. DNA of viable *Salmonella* was amplified and visually detected within 60 min and dead cells were effectively excluded. We assessed the specificity and sensitivity of the PMA-RPA-CRISPR/Cas12a assay. The results showed that the assay had a high level of specificity, with no reactions observed with other pathogens. The application of PMA has no effect on the sensitivity of RPA-CRISPR/Cas12a technology and the visibility of the fluorescence reporting system. We successfully detected viable *Salmonella* in wastewater with a minimum detection limit of 10^1^ CFU/mL. In summary, the PMA-RPA-CRISPR/Cas12a system developed in this study allows for the rapid and visual detection of viable *Salmonella* in wastewater at concentrations as low as 10^1^ CFU/mL. By integrating PMA with the RPA-CRISPR/Cas12a technology, this system offers valuable technical support for the efficient, sensitive, and clear detection of viable *Salmonella* in wastewater.

## 1. Introduction

*Salmonella* is among the most common pathogens responsible for foodborne illnesses in humans [1,2]. It is commonly found in humans and animals, primarily excreted into the environment through feces. Urban wastewater, aquaculture effluents, and medical wastewater serve as significant reservoirs and vital transmission sources for *Salmonella*. This pathogen can persist in untreated feces and wastewater for periods ranging from five months to two years [3]. Infection by *Salmonella* may cause various diseases, such as bacteremia, gastroenteritis, food poisoning, typhoid fever, paratyphoid fever, and extraintestinal focal infections. Therefore, a rapid, sensitive, and visual in situ detection of viable *Salmonella* in wastewater is essential for real-time monitoring and treatment at the source, thereby reducing the risk of infections in both humans and livestock. This approach has substantial practical implications for safeguarding human health and alleviating economic losses.

Currently, commonly used detection methods for *Salmonella* primarily include traditional culture techniques, immunological approaches, molecular biology methods, mass spectrometry, spectral analysis, and biosensor-based techniques. The traditional culture-based method is regarded as the reliable “gold standard” for detecting *Salmonella* in wastewater. However, the lengthy detection period (4 to 7 days) presents a significant limitation for the timely detection of *Salmonella* contamination, which may pose public health risks [4]. Immunological methods have limited applications due to the lack of specific targeting antibodies [4,5]. Furthermore, these methods face challenges such as cross-reactivity, antigen variation, and reduced sensitivity and specificity, which restrict their applicability [6]. Molecular biology techniques, including multi-locus sequence typing (MLST) and polymerase chain reaction (PCR), etc., require costly equipment and skilled personnel [7]. Biosensor methods, such as immunosensors [8], aptamer sensors [9], phage sensors [10], and nucleic acid biosensors [11], also encounter challenges related to high costs, hindering their widespread adoption in the market [10,12,13,14]. Therefore, rapid and point-of-care testing methods that are suitable for *Salmonella* monitoring in environmental samples such as wastewater are still needed.

To address the challenges associated with PCR technology, which requires expensive equipment and skilled personnel, numerous researchers have focused on developing isothermal amplification technologies that are cost-effective, easy to operate, and efficient in terms of time consumption [15]. Amplification (LAMP) and Recombinase Polymerase Amplification (RPA) are widely used technologies [16,17]. RPA outperforms LAMP in rapid diagnostics due to its faster amplification (10–30 min), robust performance across temperature fluctuations, simplified assay design with a single primer pair, and enhanced field-deployability through lyophilized reagent formulations [18]. In particular, RPA technology has been widely used in the real-time detection of *Salmonella* [19]. However, isothermal amplification methods are less sensitive than PCR and are more prone to false positives [20]. The CRISPR/Cas system presents a solution to the limitations of PCR and isothermal amplification techniques, positioning such systems as promising tools in molecular diagnostics. Recently, CRISPR/Cas systems (CRISPR/Cas9 [21], CRISPR/Cas12a [22], CRISPR/Cas13a [23] and CRISPR/Cas14 [24]) have been widely used to build a variety of rapid diagnostic technologies. Cas9 lacks trans-cleavage capability, rendering it unsuitable for fluorescence-based detection [25]. While Cas13a shares similar trans-cleavage properties, its RNA-targeting restriction limits applicability to DNA pathogens [26]. Therefore, among the various compatible CRISPR/Cas systems, CRISPR/Cas12a systems are appreciated by researchers for relatively shorter guide RNA, distinct protospacer adjacent motif and high specificity requirements [27,28]. For the past few years, RPA-CRISPR/Cas12a technology has been used in *Salmonella* field detection. For example, Li Liu et al. employed this technology to detect *Salmonella* in chicken and egg samples within 45 min [4]. Ji-Yun Bae et al. used the same technology to identify *Salmonella* in ready-to-eat salads, reporting a minimum detection limit of 10^1^ CFU/mL [29].

Notably, previous DNA-based molecular assays, such as PCR and RPA, were unable to distinguish between the DNA of viable and inactivated *Salmonella* cells. This limitation arises from the persistence of DNA after cell deactivation [30]. However, the risks associated with viable and inactivated *Salmonella* differ in their environmental and health impacts. Viable *Salmonella* can rapidly reproduce and transfer [31], whereas inactivated *Salmonella*, lacking gene expression and cellular activity, has a limited capacity for reproduction and virus transmission [32]. This limitation may lead to an overestimation of associated risks. The RPA-CRISPR/Cas12a system cannot distinguish between inactivated and viable *Salmonella* cells in samples, potentially resulting in false positive outcomes or an overestimation of the viable bacterial count [33]. Studies have demonstrated that RNA-based reverse transcription techniques can effectively detect viable bacteria [34]. However, the RNA extraction process is complex, requires stringent environmental conditions, utilizes toxic organic solvents, and the RNA isolated is prone to degradation [35]. In addition to this, RNA-based methods are expensive [36]. In summary, RPA offers faster reaction kinetics and enhanced field applicability, while Cas12a delivers unmatched sensitivity through its collateral cleavage-activated fluorescence detection system.

As wastewater treatment systems are designed to inactivate pathogenic microorganisms, if detection methods cannot differentiate between viable and inactivated bacteria, DNA from dead cells may be detected, resulting in false-positive outcomes. Such overestimation could exaggerate the actual presence and transmission risk of *Salmonella*, potentially misleading public health interventions. Therefore, monitoring viable *Salmonella* in treated effluent fulfills two critical objectives: (i) assessing the disinfection efficacy of the treatment process, and (ii) providing early warning and mitigating potential public health risks arising from environmental dissemination. The use of propidium monoazide (PMA) and ethidium monoazide (EMA) inhibits DNA amplification in dead cells [37]. PMA exhibits low permeability to intact cell membranes of viable bacteria but efficiently penetrates damaged membranes of non-viable cells, enabling selective DNA labeling and inactivation of dead bacteria [38]. In contrast, EMA demonstrates higher membrane permeability in viable cells, which may lead to false-positive signals. Therefore, the application of PMA pre-treatment effectively prevents the amplification of inactivated bacterial DNA, enabling qualitative and quantitative detection of viable bacteria in complex environmental samples [39]. Xu Chen et al. [40] have demonstrated that PMA pre-treatment can be effectively combined with mPCR for detection of viable *Salmonella* in environmental water. Xu Chen et al. also combined propidium monoazide (PMA) combined with competitive annealing mediated isothermal amplification (CAMP) to detect viable *Salmonella* in milk [40]. However, their detection limit remains at 10^2^ CFU/mL and requires further improvement [41]. Combining PMA pre-treatment with the RPA-CRISPR/Cas12a system shows promise in lowering the detection limit of *Salmonella* in wastewater. However, it remains uncertain whether the application of PMA impacts the sensitivity of the RPA-CRISPR/Cas12a system and the visibility of the SS-DNA reporter. The optimal treatment concentration and treatment time of PMA to combine effectively with RPA-CRISPR/Cas12a are also unknown. In this research, we developed a rapid detection system that combines PMA with RPA-CRISPR/Cas12a, providing technical support for the swift, sensitive, and visual detection of viable *Salmonella* in wastewater.

## 2. Materials and Methods

### 2.1. Preparation of Viable and Inactivated Cells

We cultured *Salmonella* (ATCC 14028) to the logarithmic stage and regulated the culture to 1.0 × 10^8^ CFU/mL. To inactivate the cells, the suspension was heated at 95 °C for 10 min. To check for complete cell inactivation, 100 µL of the heated *Salmonella* was spread onto a Luria-Bertani (LB) agar plate, with an equal volume of untreated *Salmonella* used as a positive control on a separate plate. After incubation at 37 °C for 24–48 h, no colonies were observed.

### 2.2. Wastewater Samples

The wastewater was collected in November 2024 from the regulation tank of the wastewater treatment plant located in Baitansi Village, Xiqing District, Tianjin. The basic physicochemical indicators of wastewater are presented in Table S1. To minimize the influence of microorganisms in wastewater on the results, the wastewater was sterilized prior to use by boiling the water for 20 min.

### 2.3. PMA Condition Optimization

After removing the culture medium, viable or inactivated *Salmonella* were suspended in the sterilized wastewater at a concentration of 10^8^ CFU/mL. Subsequently, PMA was added at six gradients: 0, 4, 8, 12, 16, 20, and 30 μM (final concentrations). The samples were kept in the dark for 5 min. After this incubation period, they were subjected to illumination from LED lights within the wavelength range of 465–475 nm, utilizing a LED photoreactor, as previously detailed [22]. Six time intervals—0, 5, 10, 15, 20, and 30 min—were selected to ascertain the optimal exposure time.

### 2.4. DNA Extraction, PCR and qPCR

DNA was isolated from the pellet in accordance with the guidelines provided in the Bacteria Genomic DNA kit by Omega Bio-tek (Norcross, GA, USA). Following amplification, the size and specificity of the primer sequences were confirmed through 1% agarose gel electrophoresis. The specific primer sequences are the following: F: 5′-GCCCAGCCATACGGATAAAC-3′, R: 5‘-GCGCTACCTGTCTCCTGTAT-3′.

We prepared the PCR reaction system with a total volume of 25 μL (Table S2). The amplification protocol comprised 30 cycles, starting with an initial denaturation step at 94 °C (3 min). This was followed by denaturation at 95 °C (30 min), annealing at 58 °C (0.5 min), extension at 72 °C (1.5 min), and concluding with a final extension at 72 °C (5 min).

We prepared the qPCR reaction system with a total volume of 20 μL (Table S3). The amplification protocol comprised 40 cycles. The qPCR conditions were as follows: a pre-denaturation step at 95 °C for 3 min, denaturation at 95 °C for 15 s, annealing at 55 °C for 30 s, and extension at 72 °C for 10 s.

### 2.5. RPA Primer Design and Optimization

RPA primers were developed to identify the presence of *Salmonella*, focusing on the target sequence of the *fim*Y gene. The RPA primers that were created consist of forward amplification primers designated as F (F1, F2, and F3) and reverse amplification primers labeled as R (R1, R2, and R3). The specific sequences for these primers can be found in Table 1.

We set up the RPA reaction mixture with a total volume of 10 μL (Table S4). The reaction was carried out at 37 °C for 20 min. For the negative control group, sterile water without enzymes was used as a substitute for the DNA template in the reaction.

### 2.6. Cas12a-Mediated Cleavage Analysis of the CRISPR/Cas12a System

We designed the crRNA sequence as a fragment consisting of 44 bp, utilizing RPA primer sets that exhibited high amplification efficiency and initiated immediately after the recognized PAM site (5′-TTATT-3′). The single-stranded DNA (ssDNA) reporter, tagged with a 5′ 6-FAM and a 3′ BHQ1, was synthesized by Addy Gene Technologies Ltd. (Cambridge, UK). Four crRNA were developed: crRNA1, crRNA2, crRNA3, and crRNA4, with specific sequences provided in Table 2.

After completing the RPA reaction, the products were combined to create a 30 μL CRISPR/Cas12a reaction system (Table S5). The reaction was carried out at 37 °C for 30 min.

### 2.7. Specificity and Sensitivity of the RPA-CRISPR/Cas12a System in Wastewater

#### 2.7.1. Specificity Testing

Specificity tests were conducted on a total of ten pathogenic microorganisms, including *Salmonella* Typhimurium (ATCC 14028), *Salmonella* Paratyphi B (FSCC215007 (CMCC 50094) Q-Strain 103), *Salmonella* Enteritidis (CMCC(B) 50335), *Shigella flexneri* (CMCC 51572), *Listeria monocytogenes* (CMCC(B) 54002), *Staphylococcus aureus* (CMCC 26003), *Vibrio parahaemolyticus* (ATCC 17802), *Salmonella* (CMCC 44102), *Pseudomonas aeruginosa* (ATCC 9027), *Pseudomonas fluorescens* (ATCC 13525), *Yersinia enterocolitica* (ATCC 23715), *Bacillus cereus* (ATCC 11778), *Enterococcus faecalis* (ATCC 29212), *Enterobacter aerogenes* (CMCC 45103) and *Bacillus subtilis* (CMCC 63501). The strains were added to LB broth (5 mL) and incubated overnight at 37 °C in a shaking incubator. The samples were tested according to the methods outlined in Section 2.5 and Section 2.6. For the negative control group, sterile water without enzymes was used as a substitute for the DNA template in the reaction.

#### 2.7.2. Sensitivity Testing

We adjusted *Salmonella* concentrations to 10^0^, 10^1^, 10^2^, 10^3^, 10^4^, 10^5^, 10^6^, 10^7^ and 10^8^ CFU/mL. DNA extraction was carried out using the Bacterial DNA Kit, and the extracted DNA from the different concentration gradients was tested according to the methods outlined in Section 2.4.

### 2.8. Identification of Viable Salmonella in Inoculated Wastewater and Real-World Wastewater with the PMA-RPA-CRISPR/Cas12a System

Viable and inactivated *Salmonella* suspensions, both at a concentration of 10^8^ CFU/mL, were combined with inactivated wastewater in 1.5 mL transparent PVC centrifuge tubes, with an additional tube containing only inactivated wastewater serving as a control. Two sets of samples were prepared. One set was processed according to the optimized PMA conditions described in Section 2.3, and the samples were tested using the methods outlined in Section 2.5 and Section 2.6. The second set underwent conventional PCR detection without any treatment. We collected 24 real-world wastewater samples from different regions to verify the constructed detection method. DNA extraction was carried out using the Bacterial DNA Kit, and the extracted DNA from the different concentration gradients was tested according to the methods outlined in Section 2.4. The isolated DNA was subsequently refined utilizing a PowerClean™ DNA Clean-Up Kit (MoBio Laboratories, Inc., Carlsbad, CA, USA) to reduce potential PCR inhibition. To assess DNA recovery efficiency throughout the procedure, encompassing both DNA isolation and qPCR, an internal amplification control was employed (*Escherichia coli* DH5α harboring the *CESA9* gene, which encodes cellulose synthase A9 in *Arabidopsis thaliana*), as outlined in Test S1. The integrity of the purified DNA was evaluated via agarose gel electrophoresis, and its concentration and purity were measured using a spectrophotometer (Jenway Genova, Dunmow, Essex, UK). Detailed data on DNA quality and recovery rates are provided in Table S6 of the Supplementary Materials.

### 2.9. Statistical Analysis

In the preparation of standard samples, we performed three biological replicates to thoroughly account for potential biological variability. During the actual sample testing phase, we conducted three technical replicates to minimize measurement errors and ensure the precision of the analytical process. Bar and line charts were generated using Origin 2025 (Bar charts plot the mean average value, and the error bars represent the standard deviation). The indicated significant differences (ANOVA, *p* < 0.05) were determined by analysis of variance (ANOVA), using SPSS 25.0.

## 3. Results and Discussion

### 3.1. Optimization of PMA Treatment Concentration and Exposure Time

**Figure 1:**
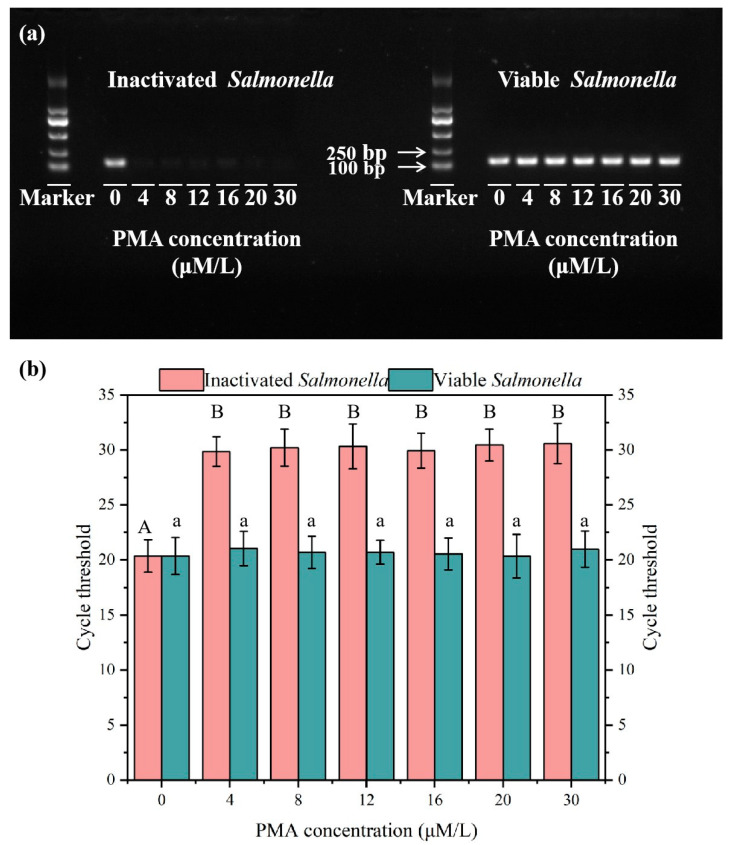
PCR amplification gel electrophoresis results (**a**) and qPCR amplification results (**b**) of inactivated or viable *Salmonella* after PMA treatment with different concentrations. Note: different capital letters indicate significant differences among inactivated *Salmonella* treatments (ANOVA, *p* < 0.05), different lowercase letters indicate significant differences among viable *Salmonella* treatments (ANOVA, *p* < 0.05). In the chart, bars represent the mean average value, and the error bar represents the standard deviation.

To ascertain the minimum necessary treatment concentration of PMA, various concentrations were tested on both inactivated and viable *Salmonella* present in wastewater to assess their effects on PCR amplification. Figure 1a illustrates that PMA treatment markedly reduced the amplification of DNA from inactivated *Salmonella*. It is evident that once the concentration of PMA surpasses 4 μM, the bands corresponding to inactivated bacteria on the agarose gel become darker, whereas those for viable bacteria remain unaffected. At a PMA concentration of 4 μM, the Ct value of untreated samples reached 30, which was significantly higher than that of the control group (PMA = 0 μM, Ct = 20.4). However, when PMA concentration exceeded 4 μM, no further increase in Ct values was observed. Notably, variations in PMA concentration did not inhibit qPCR amplification of viable *Salmonella* (ANOVA, *p* < 0.05) (Figure 1b). Consequently, the optimal PMA concentration of 4 μM was chosen because it effectively binds to the DNA of inactivated *Salmonella*, preventing their amplification while considering economic factors and minimizing reagent residue.

**Figure 2:**
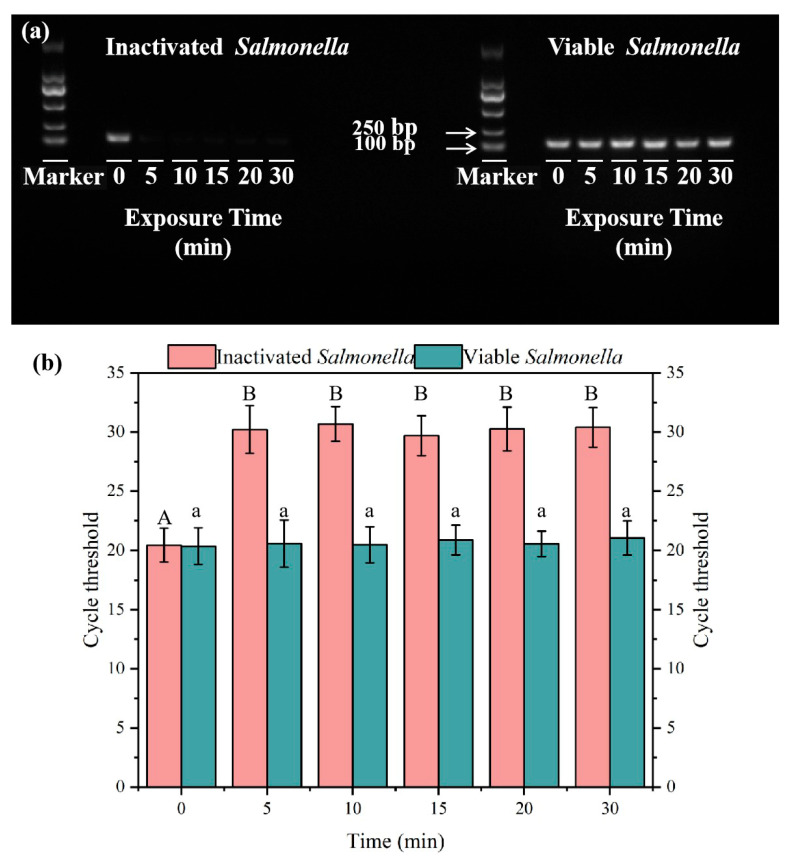
PCR amplification gel electrophoresis results (**a**) and qPCR amplification results (**b**) of inactivated or viable *Salmonella* after PMA treatment with different exposure times. Note: different capital letters indicate significant differences among inactivated *Salmonella* treatments (ANOVA, *p* < 0.05), different lowercase letters indicate significant differences among viable *Salmonella* treatments (ANOVA, *p* < 0.05). In the chart, bars represent the mean average value, and the error bar represents the standard deviation.

Utilizing the determined optimal concentration of PMA, we added 4 μM of PMA to assess how different durations of blue light exposure impact PCR amplification outcomes. As illustrated in Figure 2a, when exposed to blue light for more than 5 min, there was a significant reduction in DNA amplification of inactivated *Salmonella*, resulting in noticeably darker bands on the agarose gel. As shown in Figure 2b, exposure to blue light for 5 min significantly inhibited the DNA amplification of inactivated *Salmonella* (Ct = 20.45). When the exposure time exceeded 5 min, there was no significant change in Ct values (ANOVA, *p* < 0.05). Within the time range of 0 to 30 min, the bands corresponding to viable bacteria in the agarose gel remained unaffected, suggesting that varying durations of exposure do not impact the DNA amplification of viable *Salmonella*. Consequently, the 5-min exposure is sufficient for PMA to completely bind to the DNA of inactivated *Salmonella*. Considering experimental duration and economic factors, 5 min was selected as the optimal exposure time for the rapid detection of viable *Salmonella*. Our optimal PMA concentration is less than the previously used concentration for environmental water samples (12 μM) [41], which significantly decreases experimental costs. Selecting the appropriate concentration of PMA pretreatment can not only reduce costs and detection time but also minimize the influence of residual PMA on subsequent RPA and CRISPR reactions. The results could also provide guidance for PMA application conditions for shielding dead cells in wastewater samples.

### 3.2. Development of RPA-CRISPR/Cas12a System

To specifically identify *Salmonella*, the RPA primers were designed based on the sequence of the target gene *fim*Y within the *Salmonella* genome. The *fim*Y gene is a fimbrial gene associated with the adhesion ability of *Salmonella*, making it an ideal target for the specific identification of *Salmonella* strains. The resulting RPA primer sequences include forward amplification primers F (F1, F2, and F3) and reverse amplification primers R (R1, R2, and R3), as shown in Table 1. Various combinations of RPA amplification primers were generated. The results of gel electrophoresis indicated that the primer combinations F1/R1 and F2/R1 produced the brightest bands, demonstrating favorable amplification efficiency (Figure S1). Consequently, F1/R1 and F2/R1 were selected as the primer sets for subsequent analysis.

**Figure 3:**
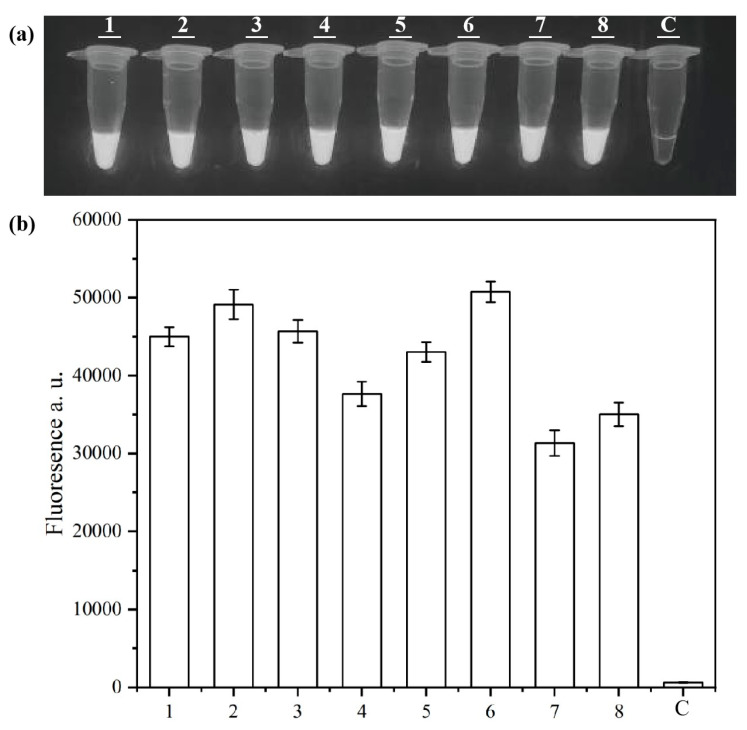
Visual detection (**a**) and fluorescence values (**b**) of RPA amplification primer and crRNA primer combination screening. Lanes 1 to 8 correspond to F1/R1-crRNA1, F1/R1-crRNA2, F1/R1-crRNA3, F1/R1-crRNA4, F2/R1-crRNA1, F2/R1-crRNA2, F2/R1-crRNA3, and F2/R1-crRNA4, and C indicates the negative control. In the chart, bars represent the mean average value, and the error bar represents the standard deviation.

Based on the optimal RPA primer pairs selected, crRNA sequences were designed into four groups: crRNA1, crRNA2, crRNA3, and crRNA4, which combined RPA primer pairs with specific crRNA sequences. Visual inspection and relative fluorescence intensity measurement confirmed that the screening results of each group showed fluorescence signals (Figure 3). Among these pairs, the fluorescence values for primer-crRNA combinations 2 and 6 were the highest, with combination 2 (F1/R1-crRNA2) showing the fastest accumulation of fluorescence, indicating superior detection performance.

### 3.3. Specificity and Sensitivity Analysis of the RPA-CRISPR/Cas12a System

**Figure 4:**
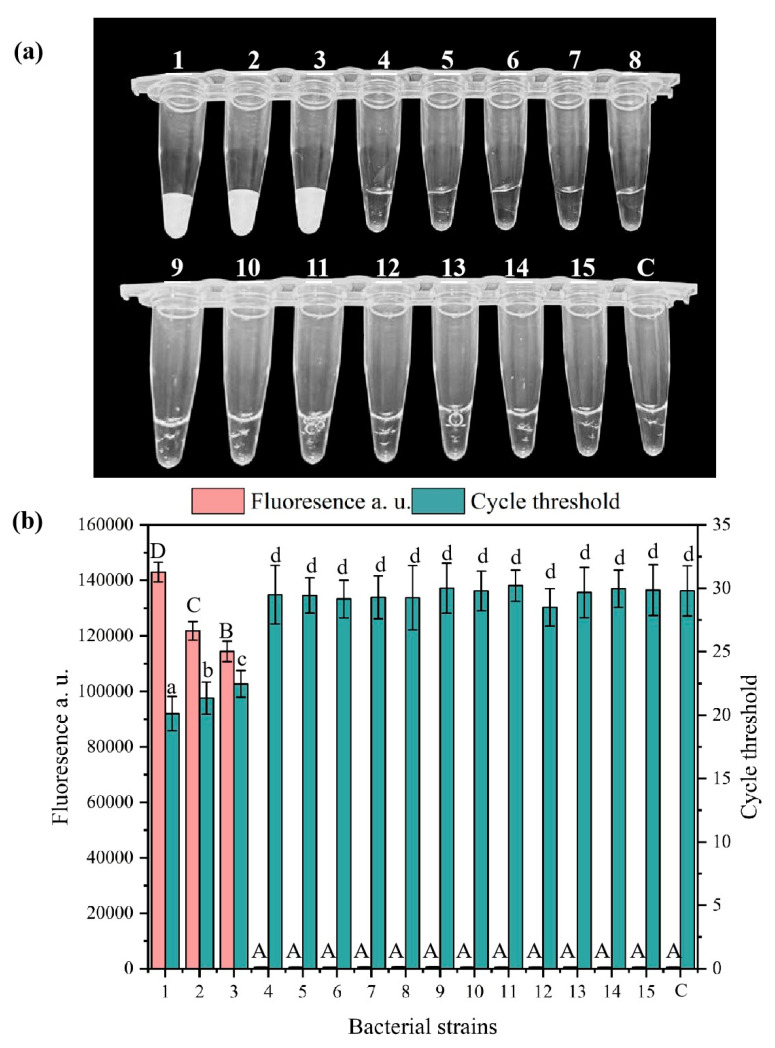
Visual detection (**a**), fluorescence and Ct values (**b**) of the fifteen common pathogens. (Lanes 1 to 15 correspond to *Salmonella* Typhimurium, *Salmonella* Paratyphi B, *Salmonella* Enteritidis, *Shigella flexneri*, *Listeria monocytogenes*, *Staphylococcus aureus*, *Vibrio parahaemolyticus*, *Escherichia coli*, *Pseudomonas aeruginosa, Pseudomonas fluorescens*, *Yersinia enterocolitica*, *Bacillus cereus*, *Enterococcus faecalis*, *Enterobacter aerogenes* and *Bacillus subtilis*, and C indicates the negative control.) Note: different capital letters indicate significant differences among fluorescence values (ANOVA, *p* < 0.05), different lowercase letters indicate significant differences among Ct values (ANOVA, *p* < 0.05). In the chart, bars represent the mean average value, and the error bar represents the standard deviation.

To verify the specificity of the RPA-CRISPR-Cas system, we tested the fluorescence reactions using fifteen bacterial pathogens that are commonly found in the environment. As shown in Figure 4, the results demonstrated that the RPA-CRISPR/Cas12a system developed displays specific reactivity towards the target strains. *Salmonella* Typhimurium, *Salmonella* Paratyphi B and *Salmonella* Enteritidis exhibited visible fluorescence reactions, as confirmed by the visual inspection and measurement of the fluorescence and Ct values. No fluorescence reactions were observed in the other bacterial samples. These findings demonstrate that the developed RPA-CRISPR/Cas12a detection method for *Salmonella* shows strong specificity and effectively differentiates *Salmonella* from the other thirteen bacterial species. Therefore, this system achieves enhanced specificity by utilizing specially designed primers for the RPA reaction in conjunction with corresponding crRNA during the CRISPR/Cas12a process.

**Figure 5:**
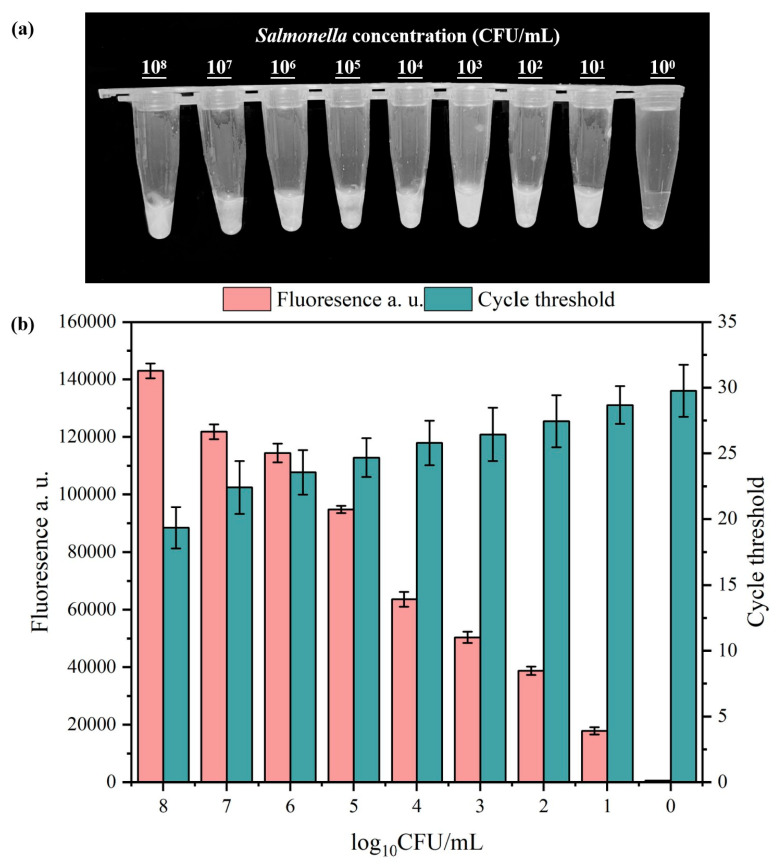
Visual detection (**a**), fluorescence and Ct values (**b**) of *Salmonella* with concentrations ranging from 10^0^ to 10^8^ CFU/mL. In the chart, bars represent the mean average value, and the error bar represents the standard deviation.

The sensitivity assessment of the developed RPA-CRISPR/Cas12a system is illustrated in Figure 5. A *Salmonella* suspension was prepared and diluted, using DNA as a template. Sterile water without enzymes was used as a negative control. Visual inspection under blue light revealed that positive fluorescence signals were clearly emitted at concentrations (10^1^ to 10^8^ CFU/mL), as confirmed by the measurement of the fluorescence and Ct values.

### 3.4. Detection of Salmonella in Wastewater Using the PMA-RPA-CRISPR/Cas12a System

**Figure 6:**
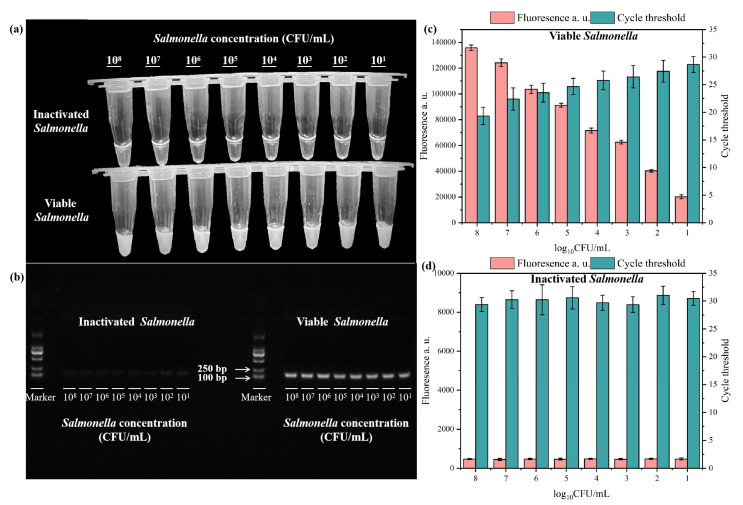
Visual detection (**a**), gel electrophoresis results (**b**), fluorescence and Ct values (**c**,**d**) of inactivated and viable *Salmonella* in wastewater with concentrations ranging from 10^1^ to 10^8^ CFU/mL. In the chart, bars represent the mean average value, and the error bar represents the standard deviation.

**Figure 7:**
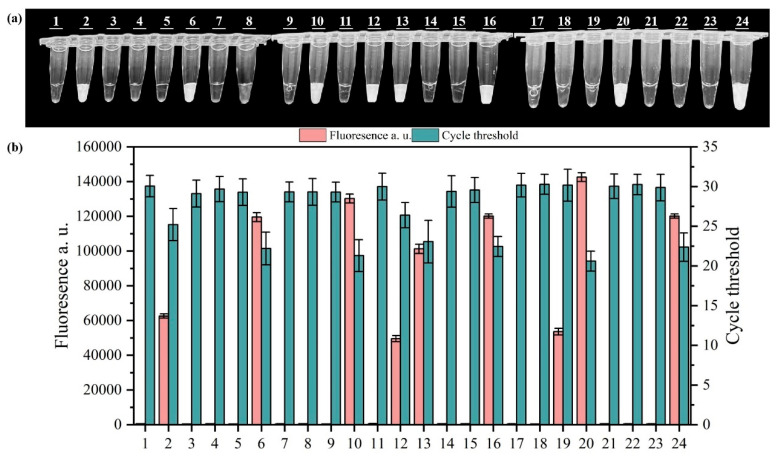
Visual detection (**a**) and fluorescence and Ct values (**b**) of test results of 24 real-world wastewater samples. In the chart, bars represent the mean average value, and the error bar represents the standard deviation.

To verify the effectiveness of the PMA-RPA-CRISPR/Cas12a system, we artificially inoculated viable or inactivated *Salmonella* into wastewater, with varying concentrations (10^1^ to 10^8^ CFU/mL). Wastewater uninoculated with *Salmonella* was used as a blank control and exhibited no signal (Figure S2a). The results were further verified using PCR and gel electrophoresis protocols (Figure S2b). As illustrated in Figure 6a,c,d, the viable *Salmonella* exhibited fluorescence signals at concentration of 10^1^ to 10^8^ CFU/mL and the inactivated *Salmonella* exhibited no signal, indicating that the developed PMA-RPA-CRISPR/Cas12a system can efficiently detect viable *Salmonella* in wastewater and shield inactivated *Salmonella*. The results were further verified using PCR and the gel electrophoresis protocol, as shown in Figure 6b. Through validation of 24 real-world wastewater samples collected from diverse sites, we observed distinct fluorescence signals in samples #2, 6, 10, 12, 13, 16, 20, and 24. These findings were further corroborated through parallel measurements of fluorescence intensity and quantitative PCR (qPCR) values (Figure 7).

In summary, the PMA-RPA-CRISPR/Cas12a system developed in this study can accurately detect viable *Salmonella* in actual samples at room temperature (37 °C) within a short time frame. The detection limit is nearly ten-fold lower than the previously developed RPA-CRISPR/Cas12a system for detecting *Salmonella* in environmental water, raw meat and milk, which could be attributed to the well-designed RPA primers and crRNA. The use of PMA did not affect the sensitivity and visibility of the RPA-CRISPR/Cas12a system. The reaction temperature of this protocol is 37 °C, which is much lower than the previously developed PMA-CAMP (competitive annealing-mediated isothermal amplification) protocol (65 °C). In summary, the method we established shows promise for the variable detection of *Salmonella* outside the laboratory, such as in onsite wastewater monitoring. We have expanded the procedure to accommodate room temperature conditions, eliminating the need for specialized amplification instruments. With the use of handheld ultraviolet equipment, on-site sewage monitoring can be achieved. By extracting DNA from the sample and minimizing contamination, we can conduct tests rapidly.

## 4. Conclusions

In this study, we developed a rapid, sensitive, and visually detectable assay that combines PMA with RPA and the CRISPR/Cas12a system for the naked-eye detection of viable *Salmonella* in wastewater. This method can be used for pathogen monitoring and early warning in the effluent of sewage treatment plants and rural sewage treatment facilities. We determined that the optimal PMA conditions are 4 μM, with a dark incubation of 5 min followed by blue light exposure for 5 min. The residual PMA did not affect the sensitivity of the RPA-CRISPR/Cas12a system and the visibility of the SS-DNA reporter. The well-designed RPA primer and the CRISPR/Cas12a system can achieve a minimum detection limit of 10^1^ CFU/mL. The entire process requires 5 min for PMA treatment, 20 min for RPA, and 30 min for CRISPR/Cas12a cleavage, significantly reducing the experimental duration and costs. Overall, the establishment of this system provides technical support for the rapid, accurate, and visual assessment of the economic, social, environmental, and health risks associated with *Salmonella* in wastewater.

## 5. Outlook

Although the PMA-RPA-Cas12A combination technology established in this study provides a feasible solution for the rapid and high-sensitivity detection of *Salmonella* in wastewater, there are still some problems such as incomplete PMA treatment and the need for portable fluorescence detection equipment for field applications. This method still faces several challenges in future practical applications. For instance, research is needed on how to quickly and easily obtain wastewater DNA without using large-scale equipment, enabling on-site detection. Additionally, the complexity of pollutants in actual wastewater raises questions about how to rapidly extract DNA from environmental samples while minimizing contaminants to reduce the impact of DNA amplification inhibitors on method accuracy. Furthermore, the binding of PMA to DNA requires blue light irradiation; however, high turbidity and color in some wastewater samples may affect the effectiveness of blue light irradiation, thereby impacting detection sensitivity. This remains an issue that requires further attention.

## Figures and Tables

**Table 1:** RPA amplification primer sequences.

Primer Set	Sequence (5′–3′)
F1	ATGTCGTGGAAAGTAACGTTTAGCTGCTG
F2	CTGGATCAGCCGAAGAAAGCTTTGCCTGTG
F3	GTGGGGAAGGTTAAGGAGGGTGATAAGTTG
R1	TATAACACAGTTTATCCGTATGGCTGGGCG
R2	TGGTATCAGATAAAACCTCCGCTATAACAC
R3	TGTCTCCTGTATTGAGGCGCTTAACCAGC

**Table 2:** crRNA Sequence.

Primer Set	Sequence (5′–3′)
crRNA1	GGGTAATTTCTACTAAGTGTAGATAGCCGGTAAACTACACGATG
crRNA2	GGGTAATTTCTACTAAGTGTAGATAAGAGGCGCCTTGCGCTAAA
crRNA3	GGGTAATTTCTACTAAGTGTAGATAAAGAAATAGCACGTCAGCA
crRNA4	GGGTAATTTCTACTAAGTGTAGATGCCGTACTGACTGGTTGATG

## Data Availability

The original contributions presented in this study are included in the article/Supplementary Material. Further inquiries can be directed to the corresponding authors.

## Supplementary Materials

The following supporting information can be downloaded at: https://www.mdpi.com/article/10.3390/microorganisms13051166/s1, Figure S1. Gel electrophoresis images of amplification products from each group in RPA primer screening. M denotes the Marker and lanes 1 to 9 correspond to the nine primer combinations: F1/R1, F1/R2, F1/R3, F2/R1, F2/R2, F2/R3, F3/R1, F3/R2, and F3/R3. C represents the negative control. Figure S2. Test results in actual wastewater artificially uninoculated. (a) PMA-RPA-CRISPR/Cas12a naked-eye observations, (b) PCR validation with PMA treatment. Table S1: The basic physicochemical indicators of the wastewater. Table S2: PCR reaction system. Table S3: qPCR reaction system. Table S4: RPA reaction system. Table S5: CRISPR/Cas12a reaction system. Table S6: A260/A280 of DNA extracted from wastewater samples (N = 3). Test S1: DNA Extraction Method and Recovery.
