# Supplementary material for: Establishment of a Sensitive and Visual Detection Platform for Viable Salmonella in Wastewater That Combines Propidium Monoazide with Recombinase Polymerase Amplification—CRISPR/Cas12a System
Source: Microorganisms. 2025 May 21;13(5):1166. doi: 10.3390/microorganisms13051166 (PMC12114456; doi:10.3390/microorganisms13051166)
Supplement: Supplementary file 1 [file microorganisms-13-01166-s001.zip › microorganisms-3545895-supplementary.pdf]

Supplementary Information for

Establishment of a sensitive and visual detection platform for viable *Salmonella* in wastewater that combines PMA with RPA- CRISPR/Cas12a system

Jiayin Liang[a,b], Xintian Sui[c], Yan Xu[a], Xiangqun Zheng[d*], Lu Tan[a,b*],

[a] Agro-Environmental Protection Institute, Ministry of Agriculture and Rural Affairs, No. 31 Fukang Road, Nankai District, Tianjin 300191, China

[b] Key Laboratory of Rural Toilet and Sewage Treatment Technology, Ministry of Agriculture and Rural Affairs, No. 31 Fukang Road, Nankai District, Tianjin 300191,

[c]Guangzhou Urban Planning&Design Survey Research Institute

[d]ChinaInstitute of Environment and Sustainable Development in Agriculture, No.12 Zhongguancun South Street, Haidian District, Beijing 100081, China

[*]Corresponding Author: E-mail addresses: tanlu_004@126.com (L Tan), E-mail: zhengxiangqun@126.com (X Zheng)

Texts Tables and Figures chapters

Test S1 DNA Extraction Method and Recovery

Table S1 The basic physicochemical indicators of the wastewater

Table S2 PCR reaction system

Table S3 qPCR reaction system

Table S4 RPA reaction system

Table S5 CRISPR/Cas12a reaction system

Table S6 A260/A280 of DNA extracted from wastewater samples (N=3)

Figure S1. Gel electrophoresis images of amplification products from each group in RPA primer screening. M denotes the Marker and lanes 1 to 9 correspond to the nine primer combinations: F1/R1, F1/R2, F1/R3, F2/R1, F2/R2, F2/R3, F3/R1, F3/R2, and F3/R3. C represents the negative control

Figure S2. Test results in actual wastewater artificially uninoculated. (a) PMA-RPA-CRISPR /Cas12a naked-eye observations, (b) PCR validation with PMA treatment

Test S1 DNA Extraction Method and Recovery

To reduce potential PCR inhibition, the isolated DNA underwent additional purification with the PowerClean™ DNA Clean-Up Kit (MoBio Laboratories, Inc.). As an internal control, the *CESA9* gene, encoding cellulose synthase A9 in the *Arabidopsis thaliana* Columbia ecotype, was employed to evaluate the efficiency of DNA recovery across the extraction and qPCR processes. Initially, the *CESA9* gene was amplified via conventional PCR and subsequently inserted into *Escherichia coli* DH5α using the *pEASY-T1* Cloning Kit (TransGen Biotech, Beijing, China). The bacterial strain was grown in Luria-Bertani (LB) liquid medium, and a 0.1 mL aliquot (OD600 = 0.631) was introduced into 100 mL of wastwater. Plasmid DNA from the *pEASY-T1* vector and genomic DNA from the bacterial strain were isolated using the *EasyPure* Plasmid Mini Prep Kit (TransGen, Beijing, China) and the *EasyPure* Genomic DNA Extraction Kit (TransGen, Beijing, China), respectively, each derived from a 1 mL culture.

The efficiency of DNA recovery (a) was determined using the following formula (1), where v represents the quantity of internal standard gene (*CESA9*) copies isolated from each wastewater sample (supplemented with 0.5 mL of the internal standard), and w denotes the number of *CESA9* copies obtained from 1 mL of the internal standard (*E. coli* DH5α) LB culture.

$$a = v \div (w/2) \qquad (1)$$

The contribution of the internal standard to the 16S rRNA count (b) was calculated using equation (2). In this equation, z represents the quantity of 16S copies isolated from 1 mL of the internal standard (*E. coli* DH5α), while a corresponds to the mean recovery rate as defined by equation (1).

$b = z \times a \qquad (2)$

To account for the influence of the internal standard, the value (b) was deducted from the total 16S gene count obtained from each wastewater sample. The integrity and efficiency of DNA extraction were assessed through agarose gel electrophoresis, with further quantification performed using a spectrophotometer (JENWAY Genova, UK). Detailed data regarding DNA quality and recovery rates are documented in Table S6 of the Supplementary Materials.

Table S1 The basic physicochemical indicators of wastewater

physicochemical indicators	pH	Ec	COD (mg/L)	TN (mg/L)	TP (mg/L)	NH3-N (mg/L)	NO3-N (mg/L)
	7.8	1.62	400	76.5	12.1	80.5	0.8

Table S2 PCR reaction system

Ingredient	System (μL)
DNA template	0.5
PCR Mix	12.5
Primer F	0.5
Primer R	0.5
DEPC-treated water	11

Table S3 qPCR reaction system

Ingredient	System (μL)
DNA template	1
qPCR Mix	10
Primer F	0.4
Primer R	0.4
DEPC-treated water	8.2

Table S4 RPA reaction system

Ingredient	System (μL)
A buffer	5.58
standard target (100 copies)	0.5
Primer F	0.4
Primer R	0.4
DEPC-treated water	2.32
B buffer	0.5

Table S5 CRISPR/Cas12a reaction system

Ingredient	System (μL)
10×cleavage buffer	3
1 μM LbCas12a nuclease	1
2 μM ssDNA reporter	1.2
RPA amplification products	5
DEPC-treated water	18.3
1 μM crRNA	1.5

Table S6 A260/A280 of DNA extracted from wastewater samples (N=3)

Sampling number	1	2	3	4	5	6	7	8
A260/A280	1.629 ±0.324	1.821 ±0.474	1.835 ±0.249	1.968 ±0.395	1.804 ±0.654	1.631 ±0.289	1.758 ±0.598	1.899 ±0.189
Recoveries (%)	65±3.3	68±3.1	60±3.4	65±2.1	61±4.5	60±3.8	68±4.7	60±2.9
Sampling number	9	10	11	12	13	14	15	16
A260/A280	1.654 ±0.325	1.762 ±0.579	1.635 ±0.267	1.914 ±0.412	1.895 ±0.598	1.835 ±0.304	1.614 ±0.415	1.588 ±0.507
Recoveries (%)	64±2.2	69±3.4	67±5.4	61±4.7	68±3.6	61±2.1	58±2.7	64±4.6
Sampling number	17	18	19	20	21	22	23	24
A260/A280	1.989 ±0.124	1.792 ±0.569	1.821 ±0.248	1.639 ±0.268	1.958 ±0.354	1.835 ±0.259	1.602 ±0.124	1.677 ±0.485
Recoveries (%)	69±3.5	69±2.7	58±4.9	67±3.1	61±3.8	68±1.4	59±3.8	64±2.6

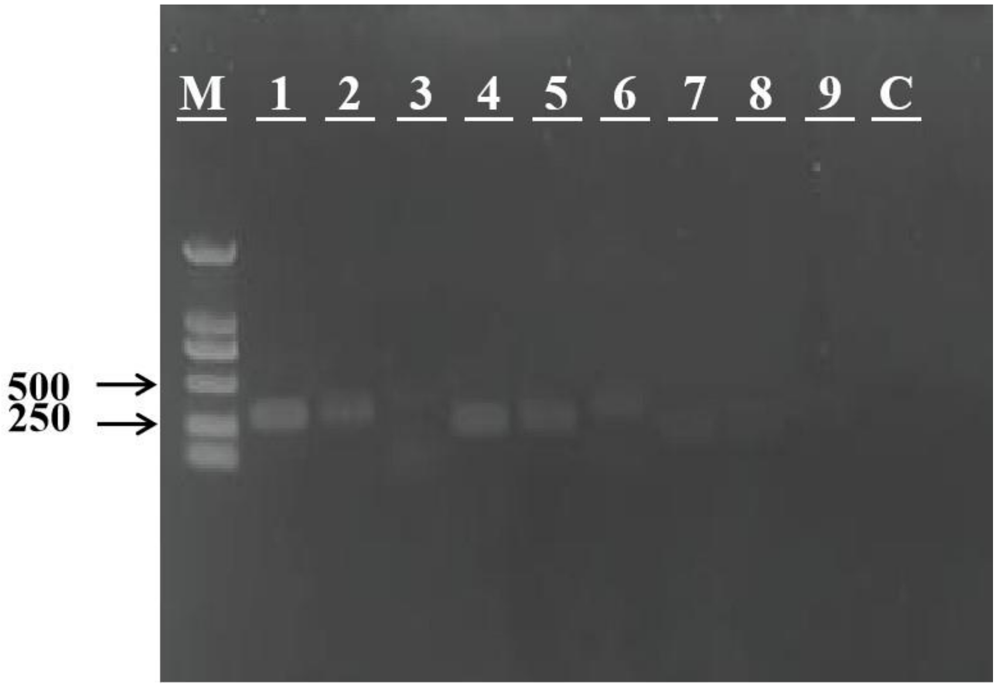

Figure S1. Gel electrophoresis images of amplification products from each group in RPA primer screening. M denotes the Marker and lanes 1 to 9 correspond to the nine primer combinations: F1/R1, F1/R2, F1/R3, F2/R1, F2/R2, F2/R3, F3/R1, F3/R2, and F3/R3. C represents the negative control.

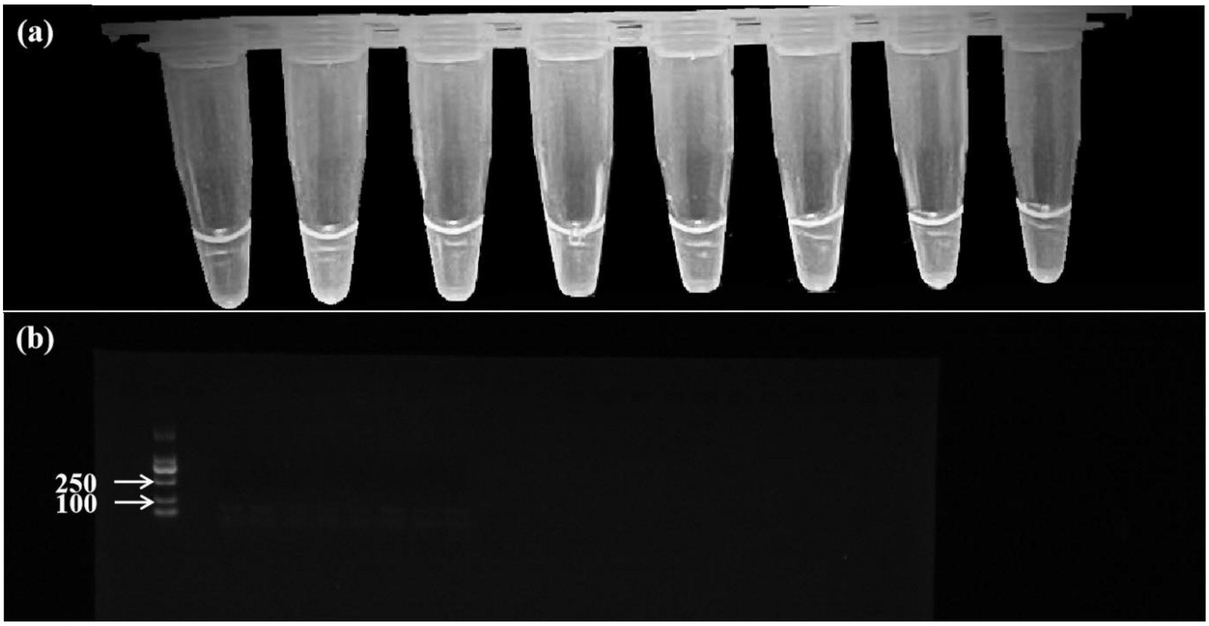

Figure S2. Test results in actual wastewater artificially uninoculated. (a) PMA-RPA-CRISPR /Cas12a naked-eye observations, (b) PCR validation with PMA treatment
